# Printable and Flexible Humidity Sensor Based on Graphene -Oxide-Supported MoTe_2_ Nanosheets for Multifunctional Applications

**DOI:** 10.3390/nano13081309

**Published:** 2023-04-07

**Authors:** Lei Ni, Xiaoyu Li, Fangkai Cai, Zhicheng Dong, Yuhong Deng, Tao Jiang, Zhengyang Su, Hao Chang, Zhongwen Zhang, Yang Luo

**Affiliations:** 1 School of Network & Communication Engineering, Chengdu Technological University, Chengdu 611730, China; 2 Engineering College of Tibet University, Lhasa 850011, China; 3 Sichuan Industial Metrology and Testing Institute, Chengdu 610100, China; dengyu.hong@outlook.com; 4 Erzhong (Deyang) Heavy Equipment Co., Ltd., Detecting &Testing Center, Deyang 618000, China; jiangtao@sinomach-he.cn

**Keywords:** humidity sensor, printable and flexible substrate, GO-MoTe_2_, respiratory monitoring

## Abstract

This study focuses on a novel humidity sensor composed of graphene-oxide (GO)-supported MoTe_2_ nanosheets. Conductive Ag electrodes were formed on PET substrates by inkjet printing. A thin film of GO-MoTe_2_ was deposited on the Ag electrode used for adsorbing humidity. The experiment’s results demonstrate that MoTe_2_ are attached to GO nanosheets uniformly and tightly. The capacitive output of the sensors with various ratios of GO/MoTe_2_ has been tested for different levels of humidity (11.3–97.3%RH) at room temperature (25 °C). As a consequence, the obtained hybrid film exhibits superior sensitivity (94.12 pF/%RH). The structural integrity and interaction of different components were discussed to afford the prominent humidity sensitivity performance. Under the bending condition, the output curve of the sensor has no obvious fluctuation. This work provides a low-cost way to build flexible humidity sensors with high-performance in environmental monitoring and healthcare.

## 1. Introduction

Humidity monitoring plays an essential role in the fields of aerospace, industry, agriculture, and even our daily lives. Resonance [1,2], optical [3,4], and electrical methods [5,6,7] have been widely investigated. For all electrical methods, electrodes have a very important effect on an interface between sensing system and analyte. There are a variety of manufacturing technologies that can be used to support the deposition and patterning of electrode materials, such as screen printing [8,9], chemical vapor deposition (CVD) [10,11], and photo lithography [12,13]. However, those methods require expensive facilities and generate hazardous waste [14,15]. Inkjet printing is a non-contact technique with flexible design, speed, and a low cost [16,17]. It does not need expensive and inflexible physical masks anymore. The desired pattern can be designed using general-purpose drawing software, and specific geometries can be implemented on the selected substrate. In view of its advantages, inkjet printing technology has some applications in the field of sensors [18,19,20].

Recently, transition metal dichalcogenides (TMDs) have excited many researchers owing to their excellent physicochemical properties and wide electronic applications [21,22,23]. Among TMDs, MoTe_2_ (molybdenum ditelluride) has attracted considerable attention in numerous fields, such as energy storage and optoelectronics, due to its high surface-to-volume ratio and favorable surface energy level [24,25]. Currently, MoTe_2_ is used as a new semiconductor material for gas and pressure sensors. Wu et al. [26] reported an ultrasensitive MoTe_2_ gas sensor for NO_2_ detection with greatly enhanced sensitivity and recovery rates under ultraviolet (UV) illumination. Seunghyun et al. [27] demonstrated a room temperature semiconductor-metal transition in thin film MoTe_2_ engineered by strain. Meanwhile, graphene oxide (GO), one of the most popular materials around the world, is an important kind of material for preparing flexible sensors, especially humidity sensors, due to its dispersibility [28], hydrophilicity [29], and large aspect ratio [30]. Zhu et al. [31] designed a fabric humidity sensor based on diamine-decorated graphene oxide/mesoporous silica nanospheres (GO–NH_2_/mSiO_2_) via screen printing. The fabric humidity sensor exhibited high sensitivity (14.8 MΩ/% relative humidity (RH)) and low hysteresis (2.71 %RH) at a humidity interval from 23% to 97%RH. Chi et al. [32] prepared a flexible humidity sensor by depositing GO film on PET substrate. Li et al. [33] fabricated a silk fabric-based respiration sensor through successive electroless plating of conductive interdigital electrodes and spray-coating of a graphene oxide (GO) sensing layer. Therefore, it is meaningful to fabricate and investigate a novel 2D sensing platform utilizing those materials with dissimilar physical properties.

In this work, a printable and flexible capacitance sensor based on GO-supported MoTe_2_ nanosheets for humidity detection was firstly demonstrated. Herein, we fabricated Ag electrodes by inkjet printing technology on a PET substrate. Then, a GO/MoTe_2_ sensing material was deposited on the electrodes by a drop-casting method. The morphology and nanostructure of the GO/MoTe_2_ nanofilm were confirmed by means of FTIR, Raman, SEM, and TEM. As a result, the sensor exhibited high response, good repeatability, and stability. At length, the underlying sensing mechanism of GO/MoTe_2_ toward humidity is also discussed.

## 2. Materials and Methods

### 2.1. Chemicals and Materials

Graphene oxide (GO, 2 mg/mL) was supplied by XFNANO Co., Ltd. (Nanjing, China). The MoTe_2_ powder was offered by Muke Nanotechnology Co., Ltd. (Nanjing, China). All other chemicals were at least of analytical grade and utilized without further purification.

### 2.2. Electrodes Fabrication

**Figure 1:**
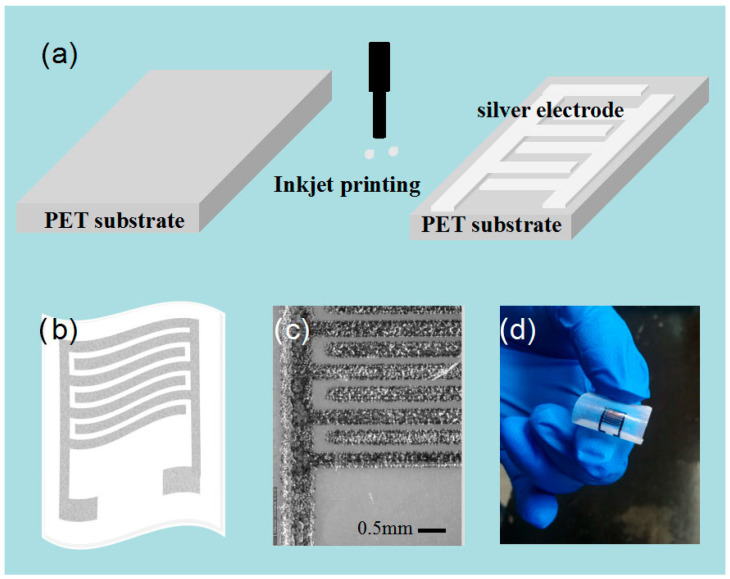
The fabrication process of Ag electrodes. (**a**) Schematic illustration of inkjet printing; (**b**) schematic diagram of flexible electrodes; (**c**) photograph of processed electrodes; (**d**) photograph of flexible substrate.

Figure 1 displays the fabrication process of Ag electrodes. The inkjet printing for Ag electrodes was performed by an EPSON R330 inkjet printer (Epson (Beijing, China) Co., Ltd.). The designed electrodes in this work were printed onto the polyethylene terephthalate (PET) substrate with the silver nanoparticle ink PrintPlus-Ink50 (JCNANO Co., Ltd., Nanjing, China). Before printing, the PET substrate was preheated to 50 °C to facilitate solvent evaporation and the solidification of the printed silver electrode [34]. The line width and the interspace between two adjacent fingers of the Ag IDEs were 500 and 250 μm, respectively, as shown in Figure 1c. The patterns were printed in 3 layers. Then, the printed Ag electrodes were rinsed with DI water and dried in a steam of nitrogen. Figure 1d exhibits the finished picture of the flexible substrate.

### 2.3. Preparation of GO/MoTe_2_ Dispersion Solution and Modification of As-Prepared Electrodes

The GO/MoTe_2_ dispersion solution was synthesized by mixing different ratios of MoTe_2_ powder into 10 mL GO solution (2 mg/mL), and then the mixture was treated with ultrasound for 1 h at 100 w. Finally, three kinds of MoTe_2_/GO nanocomposite solutions with mass ratios of 5:1, 2:1, 1:1, 1:2, and 1:5 were obtained. The obtained dispersion solution was utilized for modifying as-prepared electrodes by means of a drop casting approach. In total, 2 droplets (1 μL single droplet volume) of the mixture solution were deposited by microinjector on the working electrode area of as-prepared flexible substrate. As control groups, pure GO and MoTe_2_ were, respectively, deposited on as-prepared electrodes by the same method.

### 2.4. Instrument

SEM images were examined by FEI Inspect F50 (SEM), and TEM images were inspected by FEI Talos F200S (TEM). The Fourier transform infrared spectroscopy (FTIR) was analyzed by Nicolet IS 10. Raman measurements were performed by Horiba Scientific LabRAM HR Evolution.

The humidity sensing experiments were carried out by placing the GO/MoTe_2_ sensor in various saturated salt solutions with different relative humidity (RH) levels [35]. The capacitance response of the sensors was measured using an LCR meter (Wayne Kerr, 4100, London, UK) connected to a PC with a local area network interface. Hereon, the response and recovery times are defined as the time 90% of the final steady output signal value was reached. Sensitivity (S) is defined as S = ΔC/ΔRH (unit: pF/%RH), where ΔC is the sensor response, i.e., the change in capacitance, and ΔRH is the change in RH.

## 3. Results

### 3.1. Characterization

**Figure 2:**
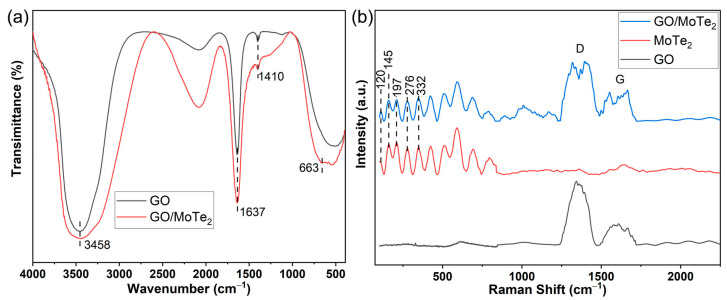
(**a**) FTIR spectra of GO and MoTe_2_/GO nano-hybrid; (**b**) Raman spectra of MoTe_2_, GO, and MoTe_2_/GO nano-hybrid.

FT-IR spectrums of the graphene oxide and MoTe_2_/GO nano-hybrid are shown in Figure 2a. The presence of different types of oxygen functionalities in GO and GO/MoTe_2_ are confirmed. The wide absorption peak at 3458 cm^−1^ is attributed to the O-H stretching vibrations between the C-OH groups and water molecules [36]. The sharp absorption peak at 1637 cm^−1^ is assigned to C=O stretching of carbonyl moiety functional groups. The sharp, tiny absorption peak at 1410 cm^−1^ is attributed to CO- carboxylic. The absorption peak at 663 cm^−1^ in nanocomposite can be assigned to MoTe_2_ [37].

Figure 2b shows Raman spectra of the MoTe_2_ film. The measurements were conducted using a 532 nm laser. Combined with the spectra of MoTe_2_, the spectra of MoTe_2_/GO illustrated the four peaks at 120 (Bu), 145 (Bu), 197 (Bg), and 276 (Ag) cm^−1^ for the film blended with MoTe_2_, affirming the crystalline layered structure of our film. The positions and relative intensities of the four peaks match well with the previous literature reports [38,39,40]. The peaks at ~1344 cm^−1^ and ~1583 cm^−1^ are defined as the D-band and G-band of graphene, respectively. The intensity ratio (ID/IG) of the two bands equals 1.5 and 1.19 for GO and MoTe_2_/GO, respectively. The ID/IG ratio can be seen as the crystallinity of graphene and a measure of defects in the skeleton structure of graphene. When the hydroxyl content is larger, the regularity of the material is worse. The GO used in this work may contain a lot of hydroxyl groups, which makes the material more hydrophilic.

**Figure 3:**
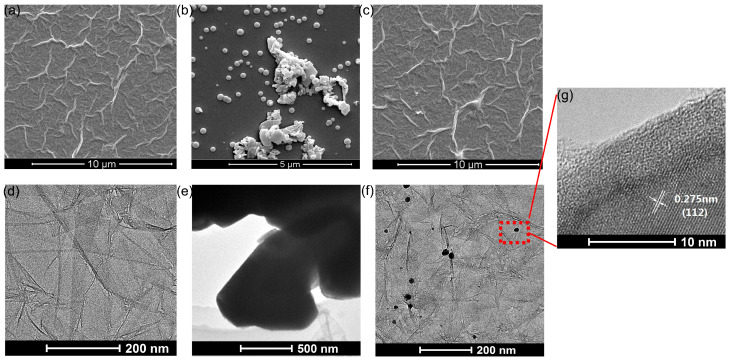
SEM (**a**–**c**) and TEM (**d**–**f**) images of GO, MoTe_2_, and GO/MoTe_2_. (**g**) HRTEM image of GO/MoTe_2_.

Figure 3 displays scanning electron microscopy (SEM) and transmission electron microscopy (TEM) images of the GO, MoTe_2_, and GO/MoTe_2_. From Figure 3a,d, it can be found that there are a large number of folds on the surface of the GO film, which are favorable for the adsorption of water molecules, and there is a small partial overlap between GO sheets. Figure 3b,e are the microstructures of nano-MoTe_2_ exfoliated from bulk ones observed by SEM and TEM. Figure 3c,f show that MoTe_2_ was well-dispersed in GO film. By comparing SEM and TEM images, it reveals that MoTe_2_ is doped between the GO sheets. In Figure 3g, the lattice fringes of the MoTe_2_ (1 1 2) plane are observed with an 0.275 nm interfacial spacing.

### 3.2. Humidity-Sensing and Flexibility Performance of the Sensor

**Figure 4:**
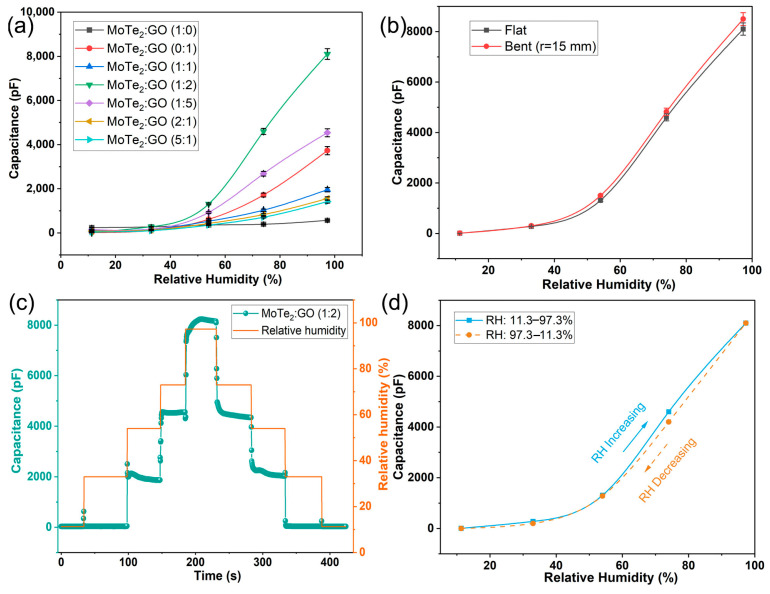
Humidity-sensing and flexibility performance of the sensor. (**a**) The humidity response of the capacitive MoTe_2_/GO sensors with different ratios from 11.3%RH to 97.3 %RH; (**b**) the capacitance changes of the sensor (MoTe_2_/GO = 1:2) as a function of relative humidity under flat and bent state; (**c**) the dynamic hysteresis loop curve of the sensor (MoTe_2_/GO = 1:2); (**d**) the typical adsorption response and desorption recovery curves of the sensor (MoTe_2_/GO = 1:2).

Humidity sensors based on MoTe_2_/GO composites with different ratios were prepared. The humidity properties of the capacitive MoTe_2_/GO sensors at 500 Hz were measured and investigated as follows, as shown in Figure 4a. The sensor with pure MoTe_2_ as the sensing material had only a 323 pF capacitance change from 11.3 %RH to 97.3 %RH. The response of the sensor with pure MoTe_2_ was remarkably lower than that of the sensor based on pure GO at different humidity conditions; however, the response of the sensor coated with MoTe_2_/GO (1:5) toward relative humidity reached 4386 pF in the whole RH range, which is about 13.5 times that of the sensor with pure MoTe_2_ and 1.3 times that of the sensor based on pure GO, respectively. These results show that the humidity sensitivity of the sensing film could be improved by doping MoTe_2_ in GO film.

The degree of improvement, however, depends on different addition amounts of MoTe_2_—too much is as bad as too little. At the outset, with the increase in the amounts of MoTe_2_ in GO film, the humidity response of sensor increases. When the mass ratio of both materials in the MoTe_2_/GO composite is 1:2, the humidity response of the sensor obtains the maximum value (capacitance change: 8093 pF), which is 25 times and 2.2 times those of the sensor with pure MoTe_2_ and the sensor with pure GO, respectively. Therefore, in the following further research, the sensor films are all in this ratio. Unfortunately, when the mass ratio of the two materials is further magnified (MoTe_2_/GO (1:1), MoTe_2_/GO (2:1), and MoTe_2_/GO (5:1)), the humidity response of sensor would actually decrease. It might be that excess MoTe_2_ could cover up not only the hydrophobic region of GO, but also part of the hydrophilic groups of GO, thus inhibiting the sensing performance improvement of the humidity sensor. It can be found that the addition of MoTe_2_ in GO thin film has little effect on its hydrophilicity under low humidity. However, under moderate and high humidity conditions, doping an appropriate amount of MoTe_2_ into GO thin films can significantly improve its hydrophilicity.

**Figure 5:**
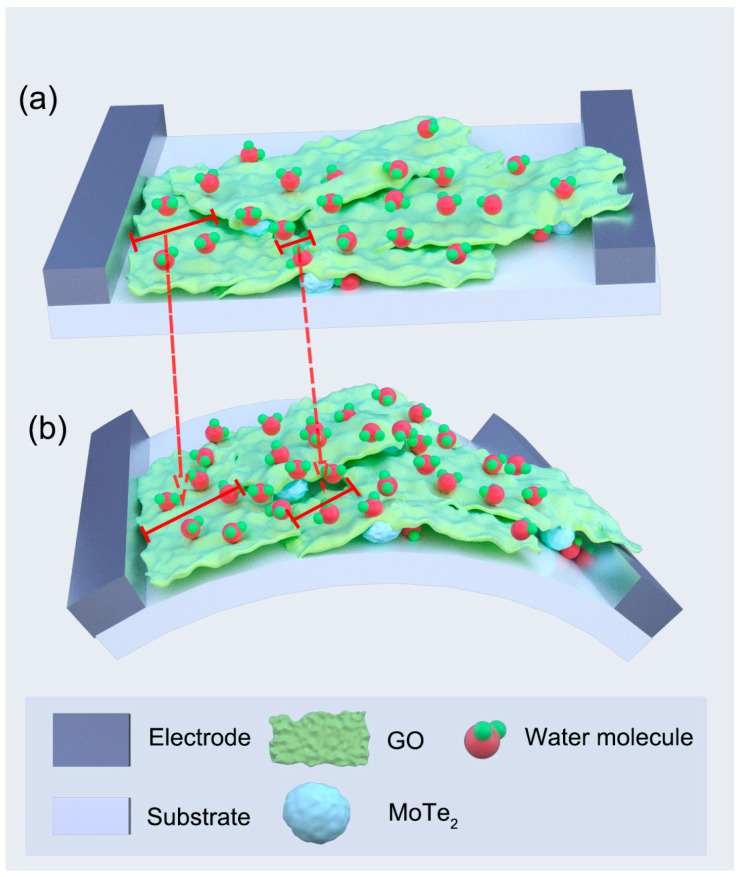
Schematic diagram of the composite film of the sensor under flat (**a**) and bending (**b**) states.

In Figure 4b, the capacitance of the humidity sensor under the bending state (r = 15 mm) is measured and, has a slight increase in comparison with that of the flat state in a humidity range of 11.3–97.3%RH. It can be explained as follows: When the composite film is coated on the flexible electrode, the GO sheets partially overlap together, as shown in Figure 3d. At the flat state (Figure 5a), a number of water molecules are adsorbed on hydrophilic groups and water absorption sites of the composite film to enhance the output performance of the sensor. However, if the humidity sensor is bent (Figure 5b) at a fixed radius, the overlap between different GO nanosheets is reduced so that the composite film has a larger surface area and can adsorb more water molecules, thus slightly increasing the capacitance of the sensor. Fortunately, the decreasing of overlap between GO nanosheets induced by bending has limited impact on the capacitance of the humidity sensor.

To evaluate the accuracy and efficiency of the humidity sensor, the hysteresis characteristic is also a significant parameter. The dynamic hysteresis loop curve is shown in Figure 4c. It can be found that the frequency shifts of the humidity sensor in the cycle of increasing humidity and decreasing humidity are approximate at each fixed humidity level. Figure 4d displays the typical adsorption response and desorption recovery characteristic curves of the humidity sensor in the range of 11.3–97.3%RH, indicating a small humidity hysteresis (~2.4%RH).

**Figure 6:**
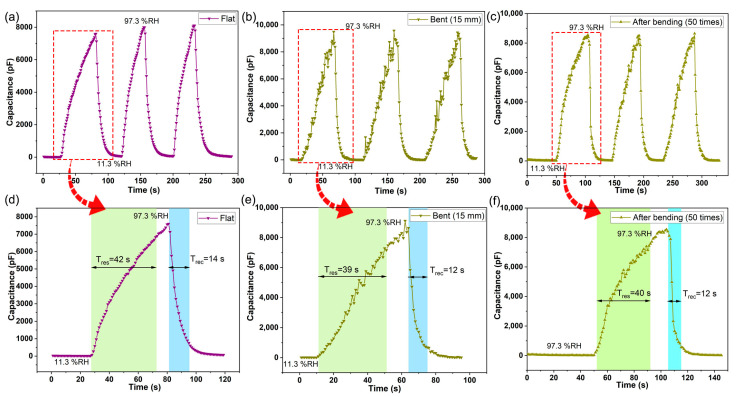
The response and recovery performance of the sensor at different states. The repeatability of the as-fabricated sensor under flat (**a**), bending (**b**), and after bending 50 times (**c**) states; the dynamic response and recovery time curves of the as-fabricated sensor under flat (**d**), bending (**e**), and after bending 50 times (**f**) states.

The repeatability of the as-fabricated sensor under both flat and bending state was studied between 11.3%RH and 97%RH. Figure 6a displays the repeatability curve of the humidity sensor for three cycles at the flat state, indicating that the sensor exhibited a good reproducibility. Furthermore, when the humidity sensor under a bending radius of 15 mm is kept in two fixed humidity levels, the sensor still maintains a good repeatability, as shown in Figure 6b. It should be noted that there will be some small disturbances in the sensor output capacitance as the humidity increases. It is possible that the bending of the as-fabricated sensor causes a small part of the GO sheet to deform so as not to be well-attached to the surface of the sensor substrate, resulting in small irregular disturbances in the output. Based on this, it can be inferred that after many bending treatments, a small part of the sensor’s sensitive film will fall off from the substrate where it is not deposited tightly, and finally the sensor output will tend to be stable. Therefore, when the bending radius is about 15 mm, after repeatedly bending the sensor 50 times, the small-amplitude interference of the output capacitance of the sensor is weakened, as shown in Figure 6c. Additionally, the as-fabricated sensor can still keep an excellent repeatability.

**Figure 7:**
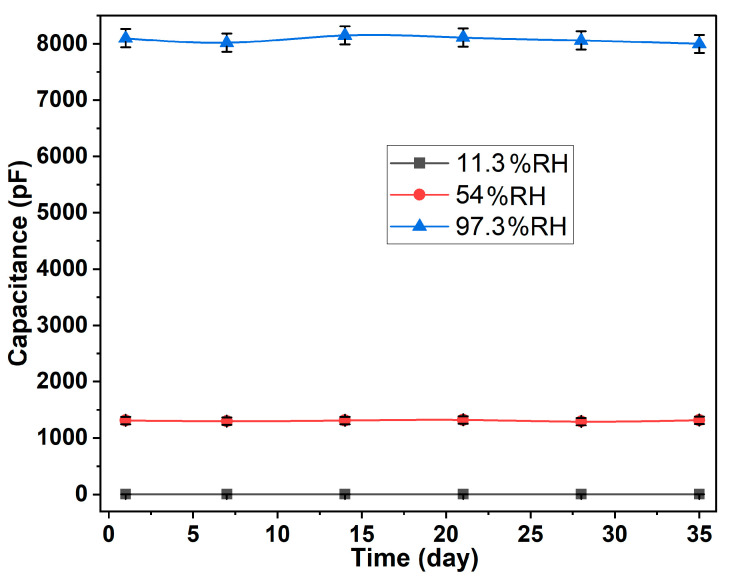
The long-term stability of the proposed sensor for 35 days.

The response and recovery time is one of the important performance indicators to evaluate the sensor. Figure 6d–f show the response and recovery time curve of the humidity sensor under different states. The response times at the flat, bending radius 15 mm, and bending radius 15 mm (after bending 50 times) are 42 s, 39 s, and 40 s, respectively, and the corresponding recovery times are 14 s, 12 s, and 12 s, respectively. It could be found that the degree and times of bending have limited influence on the response and recovery time of the sensor. Interestingly, the recovery time of the sensor is less than the response time, which is beneficial to its application in the field of wearable electronics, especially respiratory detection devices. Figure 7 shows a typical long-term stability curve of the sensor. The capacitance of the sensor was measured under various RH levels every 7 days for 5 weeks at 23 °C. The capacitance of the sensor had a slight change over time. It directly indicates that the sensor has a good long-term stability.

**Figure 8:**
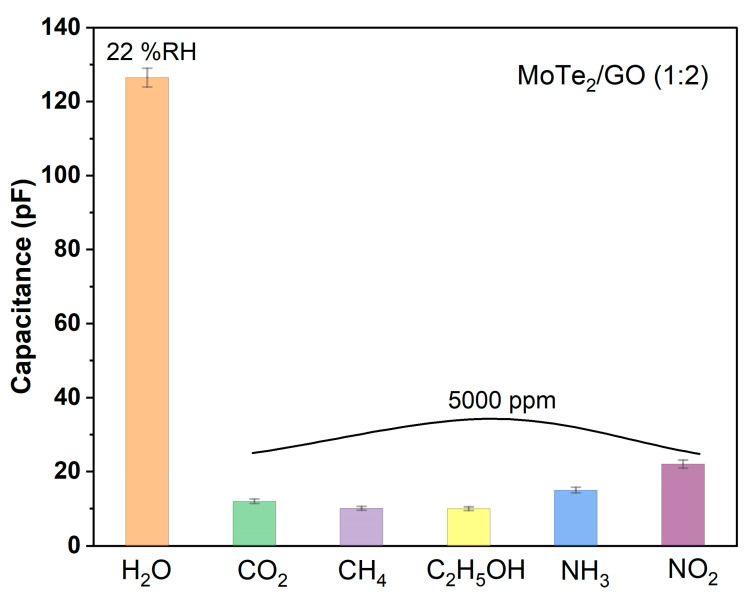
The capacitance of the proposed sensor to various gases.

Various gases, such as CO_2_, CH_4_, ethanol (C_2_H_5_OH), NH_3_, and NO_2_, have been tested to evaluate the cross-sensitivity of the sensor. The capacitance of the sensor to these gases (at 5000 ppm) was measured, as shown in Figure 8. It was obviously seen that the proposed sensor had a higher response toward humidity compared with other test gases, indicating a low cross-sensitivity to these gases for the sensor.

Table 1 lists the sensing characteristics of proposed sensor in this work to compare with the previously published flexible humidity sensors [41,42,43,44,45]. It can be found that the proposed sensor has better comprehensive performance than other listed flexible humidity sensors.

### 3.3. Humidity Sensing Mechanism

The above experimental results show that the sensor possesses excellent humidity sensing performance. Therefore, the MoTe_2_/GO film is considered as a potential humidity sensing material. The combination of MoTe_2_ nanoparticles and GO nanosheets greatly enhances the ability of humidity sensing. MoTe_2_ with a sphere nanostructure can be embedded between nanosheets to play a supporting role, resulting in a great favor to the absorption and diffusion of water molecules [46]. Moreover, the MoTe_2_/GO film can offer a higher proportion of available active sites for water molecule absorption and can accelerate the process of adsorption and desorption on the film surface owing to the larger surface specific area compared with GO [47].

**Figure 9:**
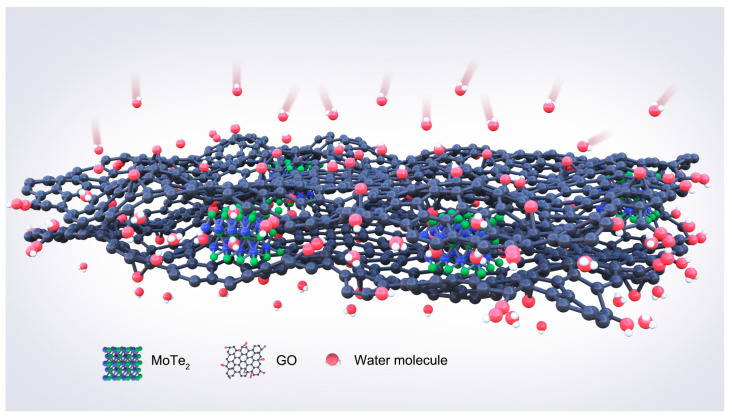
The humidity sensing mechanism schematic diagram of the sensor.

Figure 9 is a schematic diagram showing the process of water adsorption and diffusion. The capacitance changes of the sensor are caused by a large number of water molecules adsorbed on its sensitive materials. As the humidity level rises after chemisorption of hydroxyl groups takes place, physisorption occurs between hydrophilic functional groups on GO and water molecules, and forms a layer of water molecules bonded by Van der Waals attraction [48]. This increases the dielectric constant of composite materials and further enhances their capacitance change range.

### 3.4. Application to Respiratory and Non-Contact Detection

**Figure 10:**
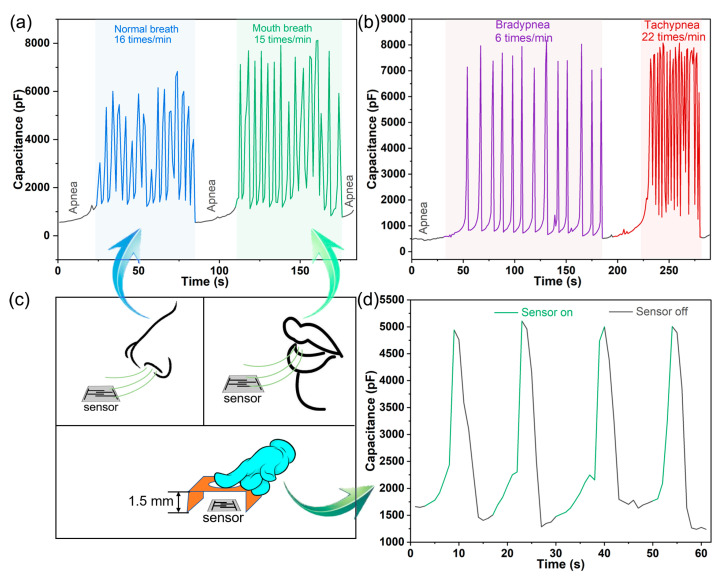
Application to respiratory and non-contact detection. (**a**) Monitoring of normal breath, breath-holding, and mouth breath; (**b**) monitoring of bradypnea and tachypnea; (**c**) schematic diagram of human respiration pattern and non-contact measurement style; (**d**) the curve of non-contact skin humidity sensing performance.

In the field of health monitoring, with the development of wearable devices, new requirements are put forward for flexible sensors, especially in respiratory monitoring. Taking into account the response and recovery characteristics of the prepared sensor, it has ability to perform real-time respiratory monitoring in a non-contact manner. The normal respiratory rate of adults in a calm state is about 12–20 times min^−1^, and the cycle of one breath is approximately 3–5 s [49]. Abnormal respiration of the frequency type includes tachypnea (respiratory rate > 20 times min^−1^) and bradypnea (respiratory rate < 12 times min^−1^). As shown in Figure 10a,c, the normal respiratory rate (a healthy 27-year-old male volunteer) is 16 times min^−1^, and the mouth breathing rate is 15 times min^−1^. Compared with a normal breathing (by nose) response, the capacitance variation range is larger due to the wetter and stronger airflow from the tester’s mouth. We simulated two kinds of abnormal breathing (tachypnea and bradypnea) and carried out a breathing experiment, as shown in Figure 10b. The experimenter imitated abnormal breathing by breathing quickly and slowly. It shows a marked distinction in the respiratory rate, but the breathing depth is at the same level. The main reason is that when breathing slowly, the exhalation time is longer and the high humidity can be maintained for a long time; during rapid breathing, the interval between two breaths is short, the humidity of the previous exhalation cannot be rapidly decreased, and the humidity of the two exhalations is superimposed, resulting in a high humidity level.

We also tested the non-contact skin humidity sensing performance, and the test method is shown in Figure 10c. Figure 10d exhibits the capacitance change of the sensor when the tester’s finger is close to or far away from the top of the sensor at 1.5 mm. When the fingertip approaches the sensor, the evaporated water molecules from the skin can be absorbed by sensing film. While the fingertip is lifted, the humidity around the sensor is quickly assimilated by the air humidity. It can be found that the sensor displays a fast response to tiny humidity variation caused by the distance between the tester’s finger and the sensing film. Thus, the non-contact sensing test has potential to be applied to contactless switching circuits.

## 4. Conclusions

In this paper, graphene-oxide (GO)-supported GO-MoTe_2_ nanosheets were deposited on the conductive Ag electrodes formed on PET substrates by inkjet printing to detect humidity. The SEM and TEM graphs demonstrate that MoTe_2_ are well-dispersed to GO nanosheets uniformly and tightly. By comparing the response of each sensor, the optimal composite ratio of materials is found out. The capacitive performance of the sensor has been tested at different states (flat, bent). The obtained hybrid film exhibits excellent sensitivity. Moreover, after bending, the response of the sensor does not change significantly, which is beneficial to its application in wearable devices. Due to its good response and recovery characteristics, the sensor also has a good performance in respiratory monitoring and non-contact measurement. The structural integrity and interaction of different components were discussed to afford the prominent humidity sensitive performance. This work proposed a low-cost and easy-to-operate preparation method of a flexible humidity sensor with high performance in environmental monitoring and medical care.

## Figures and Tables

**Table 1:** Performance comparison of the proposed sensor in this work with the previous published flexible humidity sensors.

Reference	Sensitive Material	Substrate	Sensitivity (pF/%RH)	Response/Recovery Time (s)	Humidity Rang (RH)
[41]	Polyimide	Cleancool fibers	82.44	3.5/4	6–97%
[42]	Armalcolite-PDMS	Polyimide	0.57	8.53/11.2	33–95%
[43]	P(VDF-TrFE)/GF	PET	0.27	0.8/2.5	8–98%
[44]	Graphene oxide	Paper	5.65	180/300	30–90%
[45]	WCNs (1.0 μm)	PET	23.27	50/280	7–94%
This work	MoTe2/GO	PET	94.12	39/12	11.3–97.3%

## Data Availability

Not applicable.
